# Potential of Vitamin D Food Fortification in Prevention of Cancer Deaths—A Modeling Study

**DOI:** 10.3390/nu13113986

**Published:** 2021-11-09

**Authors:** Tobias Niedermaier, Thomas Gredner, Sabine Kuznia, Ben Schöttker, Ute Mons, Hermann Brenner

**Affiliations:** 1 Division of Clinical Epidemiology and Aging Research, German Cancer Research Center (DKFZ), 69120 Heidelberg, Germany; t.gredner@dkfz.heidelberg.de (T.G.); s.kuznia@dkfz-heidelberg.de (S.K.); b.schoettker@dkfz-heidelberg.de (B.S.); u.mons@dkfz-heidelberg.de (U.M.); 2 Medical Faculty Heidelberg, Heidelberg University, 69117 Heidelberg, Germany; 3 Network Aging Research (NAR), Heidelberg University, 69117 Heidelberg, Germany; 4 Faculty of Medicine and University Hospital Cologne, University of Cologne, 50923 Cologne, Germany; 5 Cancer Prevention Unit, German Cancer Research Center (DKFZ), 69120 Heidelberg, Germany; 6 Division of Preventive Oncology, German Cancer Research Center (DKFZ) and National Center for Tumor Diseases (NCT), 69120 Heidelberg, Germany; 7 German Cancer Consortium (DKTK), German Cancer Research Center (DKFZ), 69120 Heidelberg, Germany

**Keywords:** vitamin D, fortification, cancer mortality, prevention, review

## Abstract

Meta-analyses of randomized controlled trials (RCTs) have estimated a 13% reduction of cancer mortality by vitamin D supplementation among older adults. We evaluated if and to what extent similar effects might be expected from vitamin D fortification of foods. We reviewed the literature on RCTs assessing the impact of vitamin D supplementation on cancer mortality, on increases of vitamin D levels by either supplementation or food fortification, and on costs of supplementation or fortification. Then, we derived expected effects on total cancer mortality and related costs and savings from potential implementation of vitamin D food fortification in Germany and compared the results to those for supplementation. In RCTs with vitamin D supplementation in average doses of 820–2000 IU per day, serum concentrations of 25-hydroxy-vitamin D increased by 15–30 nmol/L, respectively. Studies on food fortification found increases by 10–42 nmol/L, thus largely in the range of increases previously demonstrated by supplementation. Fortification is estimated to be considerably less expensive than supplementation. It might be similarly effective as supplementation in reducing cancer mortality and might even achieve such reduction at substantially larger net savings. Although vitamin D overdoses are unlikely in food fortification programs, implementation should be accompanied by a study monitoring the frequency of potentially occurring adverse effects by overdoses, such as hypercalcemia. Future studies on effectiveness of vitamin D supplementation and fortification are warranted.

## 1. Introduction

Recent meta-analyses of randomized clinical trials have consistently suggested a reduction of cancer mortality by approximately 13% through vitamin D supplementation [1,2]. We previously demonstrated, using cancer registry data from Germany, that vitamin D supplementation could be a very cost-effective if not cost-saving approach to reduce the burden of cancer deaths [3]. Another potentially even more cost-saving approach could be food fortification by vitamin D, a policy that has been implemented in a few countries, mainly for purposes other than prevention of cancer mortality [4,5,6,7,8]. Given that it is unlikely if not impossible that effectiveness and cost-effectiveness of vitamin D food fortification with respect to reduction of cancer mortality will ever be evaluated by randomized study designs, we aimed to evaluate the potential of vitamin D food fortification by addressing the following questions: (i) Can vitamin D food fortification achieve similar increases in vitamin D levels as vitamin D supplementation at doses that were found to be effective in reducing cancer mortality? (ii) What would be the costs for such food fortification, and how would they compare with saved costs from prevented cancer deaths?

In order to address these questions, we reviewed the literature on increases of vitamin D levels by vitamin supplementation, paying particular attention to such increases in the RCTs demonstrating a reduction in cancer mortality, the literature on increases in vitamin D levels by vitamin D food fortification, and the literature on costs of vitamin D fortification. Potential adverse effects of fortification by vitamin D overdoses will also be discussed. Finally, we used the results to compare those costs to potential savings from reduced cancer mortality, taking cancer mortality data from Germany as an example.

## 2. Materials and Methods

### 2.1. Study Selection

We searched PubMed from inception to February 2021 for studies on the effect of vitamin D supplementation and cancer mortality as well as on the effect of vitamin D supplementation and of vitamin D food fortification (with at least 4 weeks of intervention) on serum 25-hydroxy-vitamin D (25(OH)D) levels, the most commonly employed measure of vitamin D status. The search terms are listed in the Supplement. Non-English-language articles, comments, correspondences, and studies not reporting sufficient details on the key parameters of interest (baseline serum levels, daily uptake, change in serum levels, definition of intervention and control group) were disregarded. We used data from identified recent meta-analyses where possible.

### 2.2. Randomized Trials on Vitamin D Supplementation and Cancer Mortality

Three meta-analyses of RCTs on vitamin D supplementation and cancer mortality were published in 2019 (Table 1), all of which used at least PubMed and Embase databases for electronic search, used random-effects models for pooling, and obtained almost identical summary estimates of 0.87. The meta-analyses differed slightly in their time frame for search and in- and exclusion criteria and thus in included studies, numbers of participants, and cancer deaths. Apart from cancer mortality, studies also or primarily assessed other outcomes in addition to cancer mortality, such as fracture risk, type-2 diabetes, and cardiovascular disease. For our analysis, we focused on cancer mortality as outcome of interest. We focused on the studies included in the meta-analysis of Keum et al. [1] because it only included studies with at least 1 year of follow-up (actual range: 3–7 years) (Table 2). However, given the overlap in included studies and thus the similarity of results to the other meta-analyses, results would not have differed when using one of those meta-analyses instead.

### 2.3. Effect of Vitamin D Supplementation on Serum 25(OH)D Levels

Regarding the intervention effects of supplementation on serum levels of 25(OH)D, we used data from a meta-analysis of randomized trials on vitamin D (D2 or D3) supplementation in mainly Caucasian subjects over >50 years by Autier et al. [21], identified through the literature search. We also extracted data from two of the RCTs on supplementation and cancer mortality that reported on this outcome [9,10].

### 2.4. Effects of Vitamin D Food Fortification on Vitamin D Levels

For the effects of vitamin D fortified food on serum 25(OH)D levels, we selected and extracted data from published studies identified by the literature search with at least 4 weeks of intervention (actual range: 8 weeks–11 years).

### 2.5. Costs and Savings

For estimated costs of food fortification in Germany, we used data from Sandmann et al. [22]. They assumed that fortification costs comprise production costs of cholecalciferol (80% of the costs), marketing and education costs (8%), food control and monitoring costs (7%), and other program-specific recurrent production costs (5%). Sandmann et al. estimated the annual costs of cholecalciferol to be 0.11€ for 800 IU (20 µg) per person. We thus calculated proportional costs for 400 IU, 600 IU, and 1000 IU. Following the estimation of Sandmann et al. [22], we assumed processing losses of vitamin D of 41%. Further explanations on calculations of costs are provided in Supplementary Table S1. In sensitivity analysis, we assumed that costs would be higher by 20%, e.g., due to increased processing costs, or lower by 20%, e.g., due to technological advances.

We focused on savings from prevented cancer deaths and disregarded the positive impact of vitamin D on various other health outcomes, such as osteoporosis (fracture costs). In addition to preventable cancer deaths, we calculated life-years saved as preventable years of life lost (preventable YLL) and monetary savings from prevented cancer deaths as described previously elsewhere [3]. In brief, YLL were calculated as the number of cancer deaths at each age group in 2016 multiplied by further life expectancy at the midpoint of each age interval and summed across sexes and 5-year age groups. Preventable YLL were calculated as the product of total YLL with the estimated relative risk reduction achievable by vitamin D fortification (11–15%). Total saved cancer treatment costs were calculated by multiplying numbers of preventable cancer deaths with estimated average end-of-life cancer care costs. The latter have been estimated to be around 40,000€ per cancer death, as outlined in our previous study on vitamin D supplementation [3]. It should be noted that neither our previous study nor the current study aimed for a comprehensive, precise cost-effectiveness analysis or cost-savings analysis, which in our view, would not be justified given the uncertainties, variations across countries, and dynamics in several of the key factors involved (including end-of-life cancer care costs). We rather aimed to demonstrate the order of magnitude of potential effects, costs, and savings that can guide further more detailed studies and public health discussion.

## 3. Results

### 3.1. Randomized Trials on Vitamin D Supplementation and Cancer Mortality

Table 1 gives a summary of recent meta-analyses of randomized clinical trials (RCTs) of vitamin D supplementation and cancer mortality. Achieved cancer mortality reductions in the meta-analysis of Keum et al. were between 11% [11] and 17% [10], with the exception of the study of Scragg et al., which used a bolus and high monthly doses rather than a daily dosing scheme [12]. Additionally, that study was conducted in New Zealand, where UV radiation is stronger [23] and vitamin D deficiency is uncommon [24], especially when compared to Germany [25] or other European countries [26] (see Table 2 for details). Of the five studies included by Keum et al. [9,10,11,12,13], two were conducted in the USA [10,11] and UK [9,13] each. Percentages of females included ranged from 32% to 100% (median: 50.6%). All studies focused on the general population rather than vitamin-D-deficient individuals.

### 3.2. Effect of Supplementation on Serum Levels

In the two RCTs reporting on the effects of supplementation on serum levels, increases ranged from 14.6 to 30 nmol/L. Elevations of serum 25(OH)D levels with vitamin D supplementation have been consistently found in numerous further studies. In a meta-analysis among 6207 participants [21], median supplementation of 800 IU/day (range 200–10,000 IU) in 76 RCTs (46 from Europe) has been shown to increase serum levels by 1.95 nmol/L per 1 µg (40 IU) of 25(OH)D supplements per day. Median baseline levels were 45 nmol/L, and median follow-up was 8.5 (range: 4.7–36.3) months. Details on characteristics of included studies in that meta-analysis are summarized in Supplementary Table S2.

### 3.3. Effects of Vitamin D Food Fortification on Vitamin D Levels

We extracted information on the effects of intake of fortified foods on serum vitamin D levels from 10 articles [4,27,28,29,30,31,32,33,34,35] (Table 3). Intake of vitamin-D-fortified foods has been suggested to increase serum levels by approximately 1.2 nmol/L per µg (40 IU) of vitamin D consumed [36]. For example, foods fortification (milk products and soy-/cereal-based drinks with 20 IU/100 g, fat spreads with 400 IU/100 g) in Finland increased serum levels in the adult population on average from approximately 48 to 65 nmol/L (+17 nmol/L) [4]. Higher concentrations of vitamin D in foods would likely increase serum levels even more, e.g., an extra 290 IU/day from milk and bread increased serum levels by 19 nmol/L in Danish families [35], and in an Iranian study, 50 g of bread per day fortified with 1000 IU vitamin D elevated serum levels by 48 nmol/L [32].

Data on uptake of vitamin D from fortified foods and expected effects on vitamin D inadequacy were extracted from seven studies [4,29,36,37,38,39,40]. Intake of sufficiently fortified foods has been suggested to result in serum increases comparable to those of regular supplementation in a range of studies (Table 4), with increases ranging from +10 to +42 nmol/L. Thus, regular intake of at least one sufficiently fortified food product is suggested to achieve an effect on cancer mortality comparable to daily supplementation with approximately 400–1000 IU vitamin D.

### 3.4. Costs and Savings

Cancer care-related savings in the most conservative scenario would amount to more than one billion € per year if fortification reduces cancer mortality similarly as supplementation with 400 IU/day. Given the comparably low costs of fortification, this would imply net savings of almost 1 billion € per year (Table 5). Assuming higher or lower costs of fortification had only minor impact on net savings. Higher net savings of up to almost 1.4 billion € would be achievable if food fortification could be implemented in a way that 25(OH)D intake would correspond to 800–1000 IU per day. When disregarding savings from potentially prevented cancer deaths, estimated costs per life-year saved ranged from 48€ to 52€ in the main analyses. The minimum estimated costs per prevented death in sensitivity analyses was 38€, and the maximum costs were 62€.

## 4. Discussion

Higher levels of vitamin D have consistently shown to be associated with lower cancer mortality [41], and recent meta-analyses of RCTs proved effectiveness of supplementation in reducing cancer mortality [1,17]. In this study, we assessed the potential of vitamin D food fortification in preventing cancer deaths. To address this research question, we first summarized the literature on the effect of 25(OH)D fortification and serum levels of vitamin D and found that with adequate fortification, achievable serum increases are comparable to those achieved with supplementation.

Fortification resulted in serum increases of approximately 20 nmol/L in most studies and even up to 48 nmol/L in one study. This is comparable to the effect of daily intake of approximately 400 (up to ~800) IU vitamin D by supplements, as demonstrated in a range of intervention studies [21]. As indicated also by a large-scale study in Finland [4], where widespread fortification is implemented, regular intake of foods fortified with adequate amounts of vitamin D is thus expected to reduce the prevalence of vitamin D inadequacy considerably. On average, however, commonly used fortification appears to be slightly less effective than intake of equal amounts of vitamin D by supplements (+ 1.95 vs. + 1.2 nmol/L per µg (40 IU) of vitamin D). However, it seems uncertain to what extent this apparent difference reflects a true difference in effectiveness or results from the heterogeneity of studies included in the respective meta-analyses.

Observational studies from Canada [40] and Finland [4,38] demonstrated that mass fortification of milk and milk products corresponds to an additional daily intake of approximately 200 IU per day. While this is less than estimated in the above-mentioned small-scale intervention studies, serum levels in the general adult population increased to an extent that the prevalence of deficiency decreased from more than 50% to less than 10% in Finland [4], and the vast majority of adults reached intakes of ≥400 IU from diet alone. However, studies on vitamin D supplementation found the largest effects on cancer mortality with daily supplementation of 2000 IU (17%, Manson et al. [10]). Thus, even a relatively wide-spread fortification program (compared to other countries) would not suffice to reduce cancer mortality in the magnitude demonstrated by supplementation with 2000 IU/day.

Differences were also apparent in serum increases between studies focusing on supplementation and serum levels compared to the supplementation trials focusing on cancer mortality outcomes. The former found expected serum increases by 40 and 100 nmol/L for supplementation of 800 and 2000 IU/day, respectively. The latter found serum increases of only 15 and 30 nmol/L for such doses. Potential explanations might include unobserved confounders, such as differences in baseline levels, variations in trial durations (accumulation of vitamin D), differences in compliance, and differences in the extent of “contamination” of the control groups.

Despite such uncertainties, our calculations suggest that food fortification with vitamin D would likely have a high impact in reducing cancer mortality. Recent meta-analyses of randomized clinical trials demonstrated cancer mortality reductions with a daily intake of vitamin D (400 to 2000 IU/day) in the range of 11% to 17% (13% on average) [1]. There was one study each investigating the effect of 400 IU, 800 IU, and 2000 IU per day, which found cancer mortality reductions by 11%, 15%, and 17%, respectively [10,11,13]. Thus, a fortification program aiming at a daily intake of 400 IU might have the potential to reduce cancer mortality by approximately 11% but would offer potential for even higher reductions at higher doses.

Comparing costs with the assumed benefits in terms of reduced cancer mortality alone, vitamin D fortification is expected to be highly cost saving, even much more so than population-wide or targeted daily supplementation. According to the current analysis, costs of food fortification would be approximately 95% lower compared to previously estimated costs of supplementation of the population aged 50+ (3). Even when entirely disregarding savings from reduced cancer mortality, fortification would still be expected to be highly cost-effective, with costs of only approximately 50 € per life-year saved. Of note, expected savings are entirely attributable to reductions in cancer mortality and not in cancer incidence, which is not affected by vitamin D. End-stage treatment costs among patients dying from cancer are, however, more than four-fold higher than among deaths from other causes [42], implying large savings even in the absence of a reduction in cancer incidence. Regarding achievable cancer mortality reduction, fortification is thus expected to be even considerably more economic than daily supplementation with vitamin D [3], with expected effects being only slightly smaller despite estimated costs being dramatically lower. A further advantage of food fortification is that it would not require regular medication intake and would also reach subgroups of the population who may be at particularly high risk of vitamin D deficiency and less likely to take vitamin D supplements.

On the other hand, when implementing a wide-spread vitamin D fortification program, one needs to be aware of the possibility that small parts of the population may consume too much vitamin D, potentially leading to hypercalcemia and -uria by overdose [43]. A meta-analysis from 2016 [44] found that risk of hypercalcemia was elevated with supplementation (RR 1.54, 95% CI 1.09–2.18). However, the clinical relevance of this finding is unclear, as no risk increase was found for kidney stones (RR: 0.66, 95% CI: 0.41, 1.09). In addition, hypercalcemia risk is likely to be lower with supplementation of vitamin D only (without calcium). Nevertheless, measures to avoid hypervitaminosis D can and should be undertaken: first, fortified foods should have a clearly specified upper limit of added vitamin D based on average dietary habits, ensuring that even unusually large consumption of different fortified foods would not cause overdoses. This is why we modeled effects only for an intake of up to 1000 IU per day. Furthermore, representative samples of the population might be invited for regular serum measurements of vitamin D (e.g., every five years) along with a questionnaire on intake of supplements and fortified foods, which would allow studying the change in prevalence of both hypo- and hypervitaminosis D and would allow for refined amounts of vitamin D added to specific foods.

To our knowledge, no previous study comprehensively assessed the potential of vitamin D fortification of foods with respect to cancer mortality. When evaluating health impacts of vitamin D fortification of foods, other potential health effects of vitamin D also need to be considered [45]: apart from its importance for bone health, high vitamin D levels have been associated with improved cognitive function in older adults with Alzheimer’s disease [46], immune modulation [47], lower risk of acute respiratory infections, dementia, cognitive decline, and depression mainly in the elderly [48] and decreased systolic blood pressure [49], increased muscle strength [50], and positive effects on transferrin saturation and iron status [51]. Recently, positive effects of vitamin D have been also suggested with respect to COVID-19-related health outcomes [52,53,54,55].

Strengths of this study include the comprehensive literature search to assess the effect of vitamin D supplementation on serum levels and cancer mortality, on the effect of fortification of foods on serum levels, and on costs of fortification. Given the RCT-based evidence of supplementation on cancer mortality and the strong evidence on effects of fortification on serum levels being comparable to effects of supplementation, it appears plausible to assume that food fortification with vitamin D might have effects on cancer mortality comparable to supplementation.

Limitations include the lack of direct evidence on the effect of food fortification on cancer mortality, heterogeneity of studies on the effects of supplementation (different populations, doses, dosing schemes, ratio of males/females, baseline levels, etc.); uncertainty of end-of-life cancer care costs; heterogeneity of cancer-care costs regarding cancer type, stage, etc.; and lack of RCTs conducted directly in the German population. However, the prevalence of vitamin D deficiency in Germany is higher than in the U.S. [25,26,56], from where two of the RCTs originated [10,11], suggesting that cancer mortality reductions achievable in Germany might be even higher. Finally, the uncertainties in many of the key parameters, notably end-of-life cancer care costs, which vary strongly across countries and over time, precluded a precise health economic analysis. Nevertheless, given the relative magnitude of effects, costs, and savings, the key findings of high effectiveness and cost saving potential of food fortification (even without considering potential and likely other health benefits) seem to be beyond doubt.

Future well-designed studies of vitamin D fortification effects and of supplementation and cancer mortality are needed to assess the impact of supplementation and food fortification on serum levels and prevalence of deficiency in greater detail. Ideally, further RCTs of vitamin D supplementation and cancer-related mortality, paying particular attention to those cancers which contribute most to cancer mortality, should be undertaken, in particular in countries with widespread vitamin D insufficiency and deficiency, such as Germany. To study effects of fortification, a natural experiment design is conceivable in which serum levels of a country before and after introduction of fortification are compared along with a neighboring country with no change in policy as the comparator group. Additionally, the optimal amount of vitamin D added to foods needs to be carefully assessed in future studies. This includes not only the absolute amount of vitamin D added per 100 g but also the choice of foods, ideally considering both dietary habits of the population and potential losses of vitamin D during processing at a manufacturing or consumer level (e.g., when fortifying flour). Furthermore, future studies should aim for more comprehensive modeling of expected health outcomes and related costs beyond cancer.

In summary, this study suggests that vitamin D food fortification would be expected to elevate vitamin D serum levels to an extent that would be comparable to daily supplementation with 400–1000 IU, which has been demonstrated to lower cancer mortality by 11–15%. Savings from cancer prevented end-of-life cancer care costs alone would outweigh fortification costs more than 50-fold. Food fortification with vitamin D could thus be a particularly effective and economic approach to lower the increasing burden of cancer encountered by many countries [57], and its implementation should be considered in the context of national and international comprehensive cancer prevention plans. If implemented, the frequency and clinical relevance of potential adverse events by rare overdoses should be monitored in an accompanying study.

## Figures and Tables

**Table 1:** Recent meta-analyses of randomized controlled trials on vitamin D supplementation and cancer mortality.

First Author, Year, Reference	Databases Searched	Literature Searched Until	Number of Included Studies (References)	Included Participants	Cancer Deaths	Statistical Model for Pooling	RR (95% CI)
Keum 2019 [1]	PubMed, Embase	November 2018	5 [9,10,11,12,13]	75,241	1107	Random-effects	0.87 (0.79–0.96) ^1^
Haykal 2019 [14]	PubMed, Embase, CENTRAL	December 2018	5 [9,10,13,15,16]	31,163	1533	Random-effects	0.87 (0.79–0.96)
Zhang X 2019 [17]	PubMed, Embase	August 2018	7 [9,10,11,12,13,18,19]	NR	1763	Random-effects	0.87 (0.79–0.95)

Abbreviations: RR, relative risk; CI, confidence interval; NR, not reported. ^1^ The meta-analysis includes a study that used an initial bolus and high monthly doses rather than daily supplementation. Pooling results without that study (Scragg et al. [12]) results in the same point estimate of cancer mortality reduction (0.87), however.

**Table 2:** Characteristics of the 5 studies included in the meta-analysis of vitamin D supplementation and cancer mortality from Keum et al. [1], sorted by daily dose.

First Author, Year (Reference)	Country	Participants	%Women	Mean Age (Age Range) (Years)	Duration of Intervention (Years)	Follow-Up (Years)	Supplementation Dose	Baseline 25(OH)D (nmol/L)	Increase in 25(OH)D Levels, Measurement	RR (95% CI) for Cancer Mortality
Wactawski-Wende 2006 [11]	USA	N = 36,282; post-menopausal women	100	50–79	Mean 7	Mean 7	400 IU/day	Median (IQR) 42.4 (31.0–58.3)	Intervention: +12 nmol/L Control: NR	0.89 (0.77–1.03)
Avenell 2012 [13]	UK	N = 5292; previous low-trauma fracture	84.7	77 (≥70)	2–5	3	800 IU/day	Mean 38	Intervention: +24 nmol/L after 1 year Control: +6 nmol/l after 1 year	0.85 (0.68–1.06)
Trivedi 2003 [9]	UK	N = 2686; doctors	31.9	74.8 (65–85)	5	5	100,000 IU/ 4 months (≙820 IU/day)	Not measured	Vs. placebo Men: +14.6 nmol/L Women: +26.4 nmol/L,~3 weeks after intake (Sept/Oct)	0.86 (0.61–1.20)
Manson 2018 [10]	USA ^1^	N = 25,871; 71% white, 20.2% black, 4% Hispanic	50.6	67.1 (men ≥ 50, women ≥ 55)	3–6	Median (range) 5.3 (3.8–6.1)	2000 IU/day	Median 71	Intervention: +30 nmol/L Placebo: −2 nmol/L, 1 year after first dose	0.83 (0.67–1.02)
Scragg 2018 [12]	New Zealand	N = 5110; residents of Auckland	41.9	65.9 (50–84)	Median (range) 3.3 (2.5–4.2)	Median 3.3	200,000 IU initial bolus + 100,000 IU/month	Mean (SD) 66.3 (22.5)	Intervention: +56–+71 nmol/L Control: +7–+22 nmol/L	0.99 (0.60–1.64)

Abbreviations: 25(OH)D, 25-hydroxyvitamin D; RR, relative risk; CI, confidence interval; IU, international units; IQR, inter-quartile range; L, liter; SD, standard deviation; NR, not reported. ^1^ Note: The mortality reduction of 17% with 2000 IU/day in the study of Manson et al. was observed even though fortification of foods with vitamin D is already allowed in the USA to a large extent since 18 July 2016 (up to 84 IU/100 g of vitamin D3 to milk, 84 IU/100 g of vitamin D2 to plant-based beverages intended as milk alternatives, and 89 IU/100 g of vitamin D2 to plant-based yogurt alternatives) [20].

**Table 3:** Effects of vitamin D food fortification and supplementation ((v)erum/(*p*)lacebo) on serum vitamin D levels, sorted by year of publication within each food category.

First Author, Year (Reference)	Fortified Food, Year(s), (Intake)	Population, Trial Duration	Baseline Levels (nmol/L)	Follow-Up Levels	Intervention Effect (nmol/L)
Milk and milk products					
Keane 1998 [27]	Milk, June 1993–June 1994 (200 IU/day)	51 older individuals from Dublin, Ireland, 12 months	v.: 24 *p*: 25	v.: 46.25 *p*: 31.8	+15
McKenna 1995 [28]	Milk (480 IU/l), Oct/Nov 1993–March 1994	102 students + hospital personnel from Dublin, Ireland, ~4 months (Oct/Nov–March)	v: 77 *p*: 85	v: 62 *p*: 54	+16
Khadgawat 2013 [29]	Milk (600 or 1000 IU/day)	713 Indian school children, 12 weeks	11.7 (11.4–11.9 across groups)	*p*: 10.8 nmol/L; 600 IU: 22.9 nmol/L; 1000 IU: 27.7 nmol/L	+12.1 (600 IU); +16.9 (1000 IU)
Jaaskelainen 2017 ^1^ [4]	Fluid milk products and soy- and cereal-based drinks (20 IU/100 g) and fat spreads (400 IU /100 g), 2000–2011	6134 (2000) and 4051 (2011) adults representative for the Finnish population, observational pre-post design, 11 years	47.6 (men); 47.5 (women)	65.2 nmol/L (men); 65.6 nmol/L (women)	+17.6 (men); +18.1 (women)
Kruger 2019 [30]	Milk powder (600 IU/day), 2019	133 Premenopausal Chinese women living in Malaysia, 12 months	48.6	60.8 nmol/L	+12.2
Gasparri 2019 [31]	Yoghurt 2011–2018	Various (meta-analysis), N = 665, 8–16 weeks	Various	Various	+31.0
Bread					
Nikooyeh 2016 [32]	Bread (1000 IU/50 g), 2015	90 healthy individuals aged 20–60 years from Iran, 8 weeks	v: 33.9 *p*: 34.7	v: 72.9 *p*: 25.4	+48.3
Itkonen 2016 [33]	Bread, 2016 (1040 IU/day in 87 g of bread)	41 young adult women recruited from Finnish university campus, 8 weeks	v: 64.6 *p*: 66.2	v: 71.6 *p*: 66.2	+7.0
Other or several products					
Biancuzzo 2010 [34]	Orange juice (OJ), 2010 (1000 IU/237 mL of juice)	105 adults aged 18–79 from the U.S., 11 weeks	D3 in OJ: 17.9 D2 in OJ: 15.8 *p*: 19.8	D3 in OJ: 30.7 D2 in OJ: 26.4 *p*: 18.1	D2 in OJ: +14.5; D3 in OJ: +12.3
Madsen 2013 [35]	Milk and bread, 2010–2011 (median 376 IU/day in intervention vs. 88 IU in control group)	201 families in Denmark (82), 6 months	v: 73 *p*: 70	v: 63 *p*: 41	+19

Abbreviations: v, verum; *p*, placebo; IU, international units; NR, not reported; OJ, orange juice; ref, reference. ^1^ The study of Jaaskelainen et al. 2017 is (to our knowledge) by far the largest on the association between fortification and serum levels.

**Table 4:** Uptake of vitamin D, including uptake from fortified foods.

First Author, Year, Ref.	Country, Population, Age	Fortified Food(s)	Estimated Daily Average Uptake (IU)			Serum 25(OH)D Levels (nmol/L)			Prevalence of Vitamin D Inadequacy	
			Before	After	Diff.					
Khadgawat 2013 [29]	India, children aged 10–14 years	Milk	NR	NR	+600	All: +30; Boys: +30; Girls: +30			NR	
				NR	+1000	All: +42; Boys: +40; Girls: +42.5			NR	
Black 2015 [37]	Ireland, adults aged 18–64 years	Fat spreads, milk	116 (Median)	140 (Median)	+24	Assuming + 2 per 20 IU: +2.4			NR	
Raulio 2017 [38]	Finland, representative adult Finnish population	Milk products, fat spreads	Men, 25–44 y: 180	Men, 25–44 y: 444	+264	Assuming +2 per 20 IU: Men, 25–44 y: +13 Men, 45–64 y: +6.4 Women, 25–44 y: +10 Women, 45–64 y: +9.2			NR	
			45–64: 276	45–64: 452	+176					
			Women, 25–44: 132	Women, 25–44: 332	+200					
			45–64: 164	45–64: 352	+188					
Jaaskelainen 2017 [4]	Finland, ≥30 years	Milk products, fat spreads	Men: 280 Women: 280	Men: 560 Women: 480	+180 +200	48 → 65 (+ 17) Largest increases in those with previously lowest levels: +34 (<30); +24 (30−<50); +10 (≥50)			Men: 54.8% → 9.4%, Women: 56.5% → 8.9% 74% of men and 58% of women reached ≥400 IU/day from diet alone	
Black 2012 [36]	NA (meta-analysis)	NA	NA	NA	+440 per 40	+19.4 +1.2			NR	
Modelled effects of hypothetical fortification scenarios										
McCourt 2020 [39]	Ireland, adults ≥ 50 years	Mostly (93%) milk, fat spreads, cereals	Milk: +36 Fat spreads: +8			NR				
						Before	M2	M3	M4	M5
Shakur 2014 [40]	Canada, nationally representative, 51–70 years	Milk, yoghurt, cheese (uptake per serving in IU)	“Model 1”/M1: reference, no fortification, 0 M2: 50 (cheese + yoghurt), 108 (milk) M3: 150 (cheese + yoghurt + milk) M4: 270 (milk), 150 (cheese + yoghurt) M5: 270 (milk + cheese + yoghurt)			Men 80% Women 90%	70% 83%	<40% 60%	~25% 45%	15% 30%

Abbreviations: IU, International Units (40 IU = 1 µg); Diff., difference; l, liter; “→”, from … (before intervention) to … (after intervention); NR, not reported.

**Table 5:** Expected costs of and savings from vitamin D food fortification in Germany with respect to cancer mortality in 2016.

Intake of 25(OH)D by Food Fortification in IU/Day (40 IU = 1 µg) ^1^	Assumed Serum 25(OH)D Increase	Assumed Corresponding Expected Mortality Reduction	Cancer Deaths Prevented	Corresponding Savings (in Thousand €)	Costs (in Thousand €)	Net Savings (in Thousand €)	€/Life-Year Saved, Disregarding Savings
400	+20 nmol/L	11%	25,281	1,011,239	15,166	996,073	49
20% lower costs	+20 nmol/L	11%	25,281	1,011,239	12,133	999,106	39
20% higher costs	+20 nmol/L	11%	25,281	1,011,239	18,199	993,040	59
*600*	+30 nmol/L	*13%*	29,877	1,195,100	17,493	1,177,607	48
20% lower costs	+30 nmol/L	*13%*	29,877	1,195,100	13,994	1,181,106	39
20% higher costs	+30 nmol/L	*13%*	29,877	1,195,100	20,991	1,174,109	58
800	+40 nmol/L	15%	34,474	1,378,962	19,819	1,359,143	47
20% lower costs	+40 nmol/L	15%	34,474	1,378,962	15,855	1,363,107	38
20% higher costs	+40 nmol/L	15%	34,474	1,378,962	23,783	1,355,179	57
*1000*	+50 nmol/L	*15.3%*	35,164	1,406,541	22,146	1,384,395	52
20% lower costs	+50 nmol/L	*15.3%*	35,164	1,406,541	17,717	1,388,824	41
20% higher costs	+50 nmol/L	*15.3%*	35,164	1,406,541	26,575	1,379,966	62

Assuming savings of 40,000€ per prevented cancer death. Italic: Interpolated from achieved mortality reduction with lower and higher daily doses. ^1^ One µg (40 IU) of 25(OH)D is expected to increase serum levels by approximately 2 nmol/L, following the meta-analysis of Autier et al. [21].

## Data Availability

All data supporting reported results are publicly available as described in the Methods section.

## Supplementary Materials

The following are available online at https://www.mdpi.com/article/10.3390/nu13113986/s1, Table S1: Further explanations on calculations of costs for vitamin D fortification of foods in Germany; Table S2. Main characteristics of randomized trials on vitamin D supplements (adapted from Autier et al. [21]).
