# Supplementary material for: Potential of Vitamin D Food Fortification in Prevention of Cancer Deaths—A Modeling Study
Source: Nutrients. 2021 Nov 9;13(11):3986. doi: 10.3390/nu13113986 (PMC8621821; doi:10.3390/nu13113986)
Supplement: Supplementary file 1 [file nutrients-13-03986-s001.zip › nutrients-1418112-supplementary.pdf]

Table S1. Further explanations on calculations of costs for vitamin D fortification of foods in Germany.

Parameter	Estimate	Calculation details	Reference
German population size	83,100,000	–	German Federal Statistical Office [1]
Annual variable costs of food fortification with 800 IU per day per person	0.11 €	–	Sandmann et al. 2015 [2]
Variable costs of food fortification with 800 IU per day for the German population	9,141,000 €	0.11 € * 83,100,000	Based on German Federal Statitistical Office [1], Sandmann et al. 2015 [2]
Processing loss of vitamin D	41%	–	Sandmann et al. 2015 [2]
Total costs of food fortification with 800 IU per day for the German population	19,819,421 €	Annual variable costs (800 IU) * (1/(1-processing loss)) *(1/0.8)), where 1/0.8 represents +20% extra costs for marketing and education costs, food control and monitoring, other programme-specific recurrent production costs	Sandmann et al. 2015 [2]
Thereof total fixed costs	10,512,221 €	Total costs of food fortification with 800 IU per day minus variable costs	Own calculation
Total costs of food fortification with 400 IU per day for the German population	15,165,821 €	Variable costs of food fortification with 800 IU per day / 800 * 400 + total fixed costs	Own calculation
Total costs of food fortification with 600 IU per day for the German population	17,492,621 €	Variable costs of food fortification with 800 IU per day / 800 * 600 + total fixed costs	Own calculation
Total costs of food fortification with 1000 IU per day for the German population	22,146,221 €	Variable costs of food fortification with 800 IU per day / 800 * 1000 + total fixed costs	Own calculation
Variable costs of food fortification with 800 IU per day for the German population	9,141,000 €	0.11 € * 83,100,000	Based on German Federal Statitistical Office [1], Sandmann et al. 2015 [2]

Abbreviations: IU, International Units (40 IU = 1 μg).

Table S2. Main characteristics of randomized trials on vitamin D supplements (adapted from Autier et al. [3])

Baseline serum 25-hydroxyvitamin D level (ng/ml)	
Median (Range)	18.1 (4.7–36.3)
No. of intervention groups with dose of	
200– <400 IU/d	8
400– <600 IU/d	18
600– <1000 IU/d	54
1000– <2000 IU/d	14
2000– <4000 IU/d	4
≥4000 IU/d	3
No. of trials with median levels	
Dose of supplement (IU/d, Median (Range))	800 (200-10,000)
Follow-up (months)[1]	
Median	8.5
Range	1–60

[1]Time from trial inception to blood draw for in-trial measurement of serum 25-hydroxyvitamin D.

References

1. DESTATIS - Statistisches Bundesamt (German Federal Statistical Office). Bevölkerungsstand - Bevölkerung nach Nationalität und Geschlecht. Availabe online: https://www.destatis.de/DE/Themen/Gesellschaft-Umwelt/Bevoelkerung/Bevoelkerungsstand/Tabellen/zensus-geschlecht-staatsangehoerigkeit-2021.html (accessed on September 3).
2. Sandmann, A.; Amling, M.; Barvencik, F.; Konig, H.H.; Bleibler, F. Economic evaluation of vitamin D and calcium food fortification for fracture prevention in Germany. *Public Health Nutr* **2017**, *20*, 1874-1883, doi:10.1017/S1368980015003171.
3. Autier, P.; Gandini, S.; Mullie, P. A systematic review: influence of vitamin D supplementation on serum 25-hydroxyvitamin D concentration. *J Clin Endocrinol Metab* **2012**, *97*, 2606-2613, doi:10.1210/jc.2012-1238.
